# Schnyder Corneal Dystrophy in an Adolescent: A Case Report With Multimodal Imaging

**DOI:** 10.7759/cureus.89786

**Published:** 2025-08-11

**Authors:** Jan Bombuy Gimenez, Justyna Izdebska, Jacek P Szaflik

**Affiliations:** 1 Department of Ophthalmology, Medical University of Warsaw, Warsaw, POL

**Keywords:** anterior segment optical coherence tomography (as-oct), confocal microscopy, corneal opacity, crystalline deposits, schnyder corneal dystrophy, ubiad1 gene

## Abstract

Schnyder corneal dystrophy (SCD) is a rare, autosomal dominant, bilateral corneal dystrophy characterized by progressive deposition of cholesterol and phospholipids within the central corneal stroma. The condition is associated with pathogenic variants in the *UBIAD1* gene, responsible for lipid metabolism. We report the case of a 17-year-old female presenting with progressive bilateral visual deterioration. Comprehensive ophthalmological examination, including slit-lamp biomicroscopy, in vivo confocal microscopy, and anterior segment optical coherence tomography (AS-OCT), was performed. The examination revealed bilateral stromal haze and central corneal crystalline deposits characteristic of SCD. The diagnosis required careful differentiation from other conditions presenting with corneal opacities or crystalline deposits, including other corneal dystrophies and systemic disorders affecting lipid metabolism. Early recognition through characteristic slit-lamp findings and multimodal imaging is decisive for appropriate management and monitoring of disease progression. Treatment options range from optical correction in early stages to phototherapeutic keratectomy, with corneal transplantation reserved for advanced cases. This case highlights the diagnostic value of combining clinical examination with in vivo confocal microscopy and AS-OCT in establishing the diagnosis of SCD, even when genetic testing is not performed.

## Introduction

Schnyder corneal dystrophy (SCD) is a rare, autosomal dominant, bilateral corneal dystrophy characterized by progressive deposition of cholesterol and phospholipids within the central corneal stroma [1]. Van Went and Winbaut first described it in 1924, and later in 1929, Schnyder reported a familial case series involving three generations [2]. The condition is associated with pathogenic variants in the UbiA prenyltransferase domain-containing 1 (*UBIAD1*) gene, which is involved in lipid metabolism through the biosynthesis of vitamin K2 and coenzyme Q10 [1,3]. SCD typically begins in the first or second decade of life with the development of central corneal haze and crystalline deposits, which may initially be asymptomatic and only detectable on slit-lamp examination or imaging [4,5]. As the disease progresses, corneal opacification may increase, potentially requiring corneal transplantation.

We present the case of a 17-year-old female with bilateral crystalline corneal deposits and visual impairment, in whom the clinical findings and multimodal imaging were consistent with SCD.

## Case presentation

A 17-year-old female patient presented with a 10-month history of progressive visual deterioration, more marked in the left eye. She was born prematurely and underwent laser photocoagulation due to retinopathy of prematurity. She had no comorbidities or relevant systemic medical history. There was no family history of any ocular disease. She had been receiving care from a local ophthalmologist, where she was reportedly prescribed spectacles and cycloplegic eye drops. Refraction examination revealed myopia and myopic astigmatism. Cycloplegic refraction was −1.50 D/−2.00 D × 147° in the right eye and −2.00 D/−2.00 D × 003° in the left eye. On examination, uncorrected distance visual acuity was 20/100 (logMAR 0.7) in the right eye and 20/200 (logMAR 1.0) in the left eye, while best-corrected visual acuity reached 20/25 (logMAR 0.1) with −2.50/−1.50 × 150° in the right eye and 20/50 (logMAR 0.4) with −4.00/−0.50 × 135° in the left eye. Near visual acuity was 0.5 (on the Snellen chart) in both eyes with the same correction. Intraocular pressure was within normal limits bilaterally.

**Figure 1:**
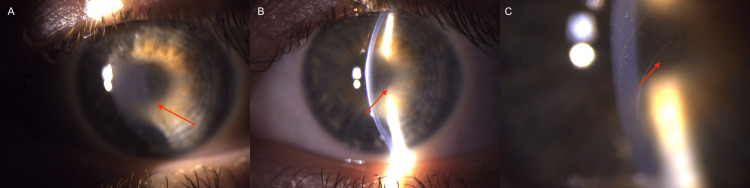
Slit-lamp photographs showing characteristic corneal findings in Schnyder corneal dystrophy. Slit-lamp images of the right eye (a-c) demonstrate central corneal crystalline opacities typical of Schnyder corneal dystrophy (red arrows). The peripheral cornea remains clear. Deposits at higher magnification (c).

Slit-lamp examination revealed central corneal crystalline deposits with mild stromal haze consistent with SCD. The peripheral corneal stroma was transparent without any sign of pathology. The anterior chambers were shallow but optically clear. The pupils were round and reactive, without signs of posterior synechiae (Figure 1). The posterior segments of the eyes were inaccessible on ophthalmological examination due to corneal opacity. In this case, the progressively limited view of the fundus posed an additional challenge in monitoring retinal changes associated with retinopathy of prematurity.

**Figure 2:**
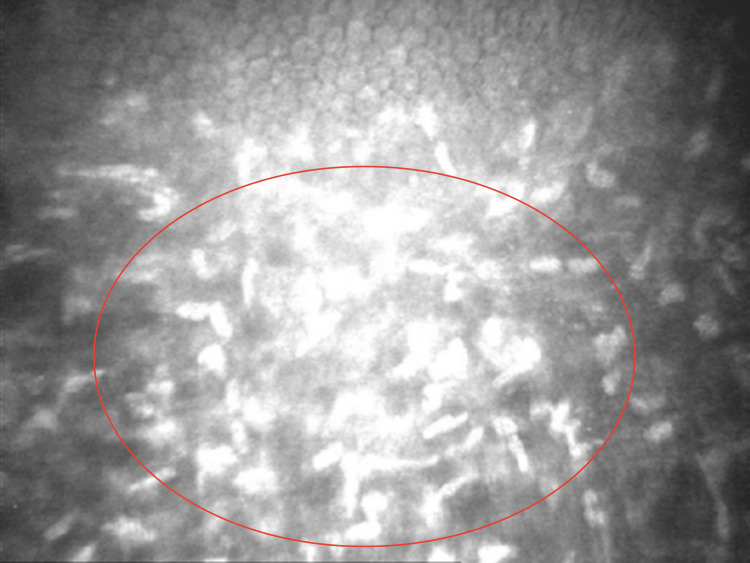
In vivo confocal microscopy image showing stromal deposits in Schnyder corneal dystrophy. In vivo confocal microscopy reveals hyperreflective, crystalline deposits within the anterior stroma of the cornea.

**Figure 3:**
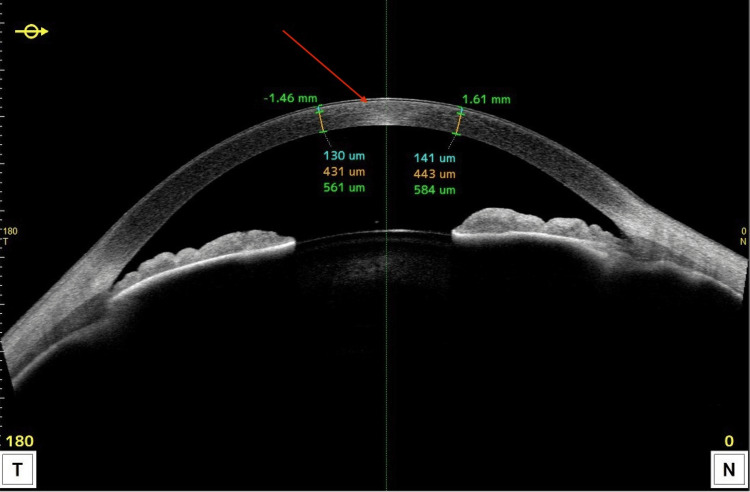
Anterior segment optical coherence tomography (AS-OCT) image showing anterior stromal changes in Schnyder corneal dystrophy. AS-OCT reveals a hyperreflective band of pathological deposits within the anterior stroma (red arrow), extending to a depth of approximately 141 µm. The total central corneal thickness measures 584 µm.

Further diagnostic evaluation included in vivo confocal microscopy (CONFOSCAN, Nidek Technologies) and anterior segment optical coherence tomography (AS-OCT) (CASIA2, Tomey). Confoscan imaging revealed a hyperreflective haze in the anterior stroma, while the surrounding stromal tissue appeared structurally normal. The corneal epithelium was of normal structure and thickness. Endothelial cells were not visualized in the central cornea; in the peripheral areas, the endothelium was clearly visible and showed normal morphology, with an estimated cell density of approximately 2750 cells/mm² in both eyes (Figure 2). AS-OCT confirmed the presence of anterior stromal haze extending to a depth of approximately 100-140 µm. The astigmatism pattern was regular, and central corneal thickness measured 565 µm in the right eye and 584 µm in the left eye (Figure 3).

## Discussion

Although the clinical presentation was characteristic of SCD, accurate diagnosis required careful differentiation from other conditions that may present with similar clinical manifestations, such as corneal opacities or crystalline deposits, including lattice corneal dystrophy (LCD), Bietti crystalline dystrophy (BCD), Fish eye disease, lecithin-cholesterol acyltransferase (LCAT) deficiency, and Tangier disease.

LCD is an autosomal dominant stromal dystrophy caused by a *TGFBI* gene mutation, characterized by branching, linear amyloid deposits within the corneal stroma [6]. Both LCD and SCD present bilaterally and may appear early in life, but LCD typically features recurrent corneal erosions and linear opacities rather than the central, discoid, crystalline stromal deposits seen in SCD. Corneal sensitivity is often reduced in LCD, which is uncommon in SCD.

BCD is an autosomal recessive retinal disorder associated with a *CYP4V2* gene mutation. It manifests with crystalline deposits in the retina and sometimes in the peripheral cornea, along with progressive chorioretinal atrophy [7]. In contrast to SCD, BCD primarily affects the posterior segment, and the crystalline deposits are not confined to the central cornea.

Fish eye disease (partial LCAT deficiency) is characterized by severe high-density lipoprotein (HDL) deficiency with systemic manifestations and progressive bilateral corneal opacification due to unesterified cholesterol deposition. The stromal haze in Fish eye disease is diffuse and lacks the crystalline features typical of SCD [8].

Complete LCAT deficiency also leads to diffuse stromal opacification, along with renal disease and anemia [8]. It is differentiated from SCD by the presence of systemic involvement and absence of discrete central crystalline deposits.

Tangier disease, caused by an *ABCA1* gene mutation, presents with extremely low HDL levels, peripheral neuropathy, hepatosplenomegaly, and corneal opacification that does not affect vision. Similar to Fish eye disease, the corneal changes are non-crystalline and diffuse [9]. Systemic features and a markedly abnormal lipid profile distinguish Tangier disease from SCD.

In contrast to all of the conditions mentioned above, SCD is limited to the cornea, with central stromal cholesterol and phospholipid accumulation, often accompanied by non-crystalline stromal haze, and is typically not associated with any systemic symptoms [10]. In imaging studies, AS-OCT typically reveals diffuse anterior stromal hyperreflectivity, often extending into the mid and posterior stroma, with the most pronounced changes observed in the anterior layers. Central corneal thickness generally ranges from 507 to 635 μm. In vivo confocal microscopy demonstrates scattered hyperreflective deposits throughout the epithelium and stroma, frequently accompanied by rare or undetectable keratocyte nuclei and thinned subepithelial nerve fibers, while the overall epithelial architecture remains preserved [11]. In the presented case, these characteristic findings were observed in both modalities, supporting the diagnosis. However, genetic testing for *UBIAD1* mutations, which would provide molecular confirmation of the diagnosis, was not performed in this case. The patient was referred for genetic testing, but declined to undergo the examination.

According to the literature, SCD most commonly becomes clinically apparent in the third decade of life, when patients begin to experience noticeable visual impairment. This symptomatic phase often coincides with the appearance of arcus lipoides in the third decade and progressive mid-peripheral stromal haze by the late fourth decade [4], even though structural changes may be detectable earlier. In the present case, the diagnosis was established in adolescence, which emphasizes the importance of early recognition of characteristic clinical and imaging findings. Early diagnosis of SCD is essential for visual prognosis and for monitoring of disease progression. Progressive central stromal haze and lipid deposition can significantly impair visual acuity and reduce quality of life. Moreover, early identification of the disease enables the prompt implementation of appropriate therapeutic strategies.

The management of SCD primarily depends on the severity of visual impairment and the extent of stromal opacification. In the early stages of the disease, treatment typically involves refractive error correction with glasses or contact lenses to improve visual acuity. However, as the disease progresses, central stromal haze and lipid accumulation can significantly reduce visual function. Excimer laser phototherapeutic keratectomy (PTK) is then recommended to remove superficial deposits and temporarily improve corneal clarity, although recurrence is common. Based on our own experience, PTK provides mild-to-moderate visual improvement and is associated with a prolonged and complex healing process. During the healing phase, the use of therapeutic (bandage) contact lenses or amniotic membrane transplantation is often required. In more advanced cases, corneal transplantation becomes necessary. Deep anterior lamellar keratoplasty (DALK) is the preferred surgical option when the endothelium is preserved, as it allows for selective replacement of the affected stroma while minimizing the risk of graft rejection. Penetrating keratoplasty may be considered in cases involving deeper stromal layers, in the event of DALK graft failure, or in cases of recurrent corneal involvement. While no medical therapies currently target the underlying metabolic defect, ongoing research into the role of *UBIAD1* mutations may offer future opportunities for targeted or gene-based treatments [3].

## Conclusions

This article presents a case study of an adolescent patient with a history of retinopathy of prematurity, who was diagnosed with SCD. The report emphasizes the importance of slit-lamp examination and multimodal imaging, including in vivo confocal microscopy and AS-OCT, in identifying characteristic SCD features, despite the subtle clinical presentation. The diagnosis at a young age allows appropriate management and monitoring of disease progression. As the cornea becomes more opacified and visual acuity decreases, suitable treatment must be tailored to the stage of disease, ranging from optical correction in early stages to surgical intervention in more advanced cases. PTK, where superficial crystalline deposits are removed, and corneal transplantation, as DALK, remain the treatments of choice in visually significant cases. Further research into targeted genetic therapies is needed to achieve lasting therapeutic solutions for patients with SCD, as none of the current surgical methods ensure complete resolution of the disease or prevent recurrence.
